# Macroporous silicon for high-capacitance devices using metal electrodes

**DOI:** 10.1186/1556-276X-9-473

**Published:** 2014-09-05

**Authors:** Didac Vega, Jordi Reina, Ferran Martí, Ramón Pavón, Ángel Rodríguez

**Affiliations:** 1 Micro and Nanotechnology Research Group (MNT), Electronic Engineering Department (EEL), Universitat Politècnica de Catalunya (UPC), c/Jordi Girona 1-3, Campus Nord Mod. C4, 08034 Barcelona, Spain

**Keywords:** Macroporous silicon, Porous silicon, Electrochemical etching, Electrodeposition, Electro-deposition, Electroplating, High-density capacitors, Energy storage

## Abstract

In this paper, high-capacity energy storage devices based on macroporous silicon are demonstrated. Small footprint devices with large specific capacitances up to 100 nF/mm^2^, and an absolute capacitance above 15 μF, have been successfully fabricated using standard microelectronics and MEMS techniques. The fabricated devices are suitable for high-density system integration. The use of 3-D silicon structures allows achieving a large surface to volume ratio. The macroporous silicon structures are fabricated by electrochemical etching of silicon. This technique allows creating large structures of tubes with either straight or modulated radial profiles in depth. Furthermore, a very large aspect ratio is possible with this fabrication method. Macroporous silicon grown this way permits well-controlled structure definition with excellent repeatability and surface quality. Additionally, structure geometry can be accurately controlled to meet designer specifications. Macroporous silicon is used as one of the electrodes over which a silicon dioxide insulating layer is grown. Several insulator thicknesses have been tested. The second capacitor electrode is a solid nickel filling of the pores prepared by electroplating in a low-temperature industry standard process. The use of high-conductivity materials allows reaching small equivalent series resistance near 1 Ω. Thanks to these improvements, the presented devices are capable of operating up to 10 kHz.

**PACS:**

84.32.Tt; 81.15.Pq; 81.05.Rm

## Background

The fast-growing portable and embedded device market has imposed increasing concerns in energy storage. Design aspects of current electronic devices demand higher integration of components, being either on printed circuit boards (PCB), on package or ultimately on die. After taking into account the devices’ interfaces to the outer world, the designer faces severe constraints to fit energy storage elements such as batteries, inductors and capacitors and meet the desired weight, size, shape and performance goals for the intended application. This is especially important for the consumer market, which demands high-performance computing for long operating periods but keeping size and weight to a minimum.

Primary energy sources have usually been batteries [1]. In particular, lithium-based batteries have dominated the landscape for advanced energy storage devices, allowing to reach gravimetric energy densities above 100 W/kg. The key to such large capacities is the use of electrochemical reactions. Nevertheless, such devices have slow reaction times due to diffusion kinetics, which causes a dramatic reduction in capacity when charged/discharged rapidly [1,2]. Other important aspects to take into account are the limited charging cycles that the active material can endure and the drop in performance when scaling down thin-film batteries [1]. Using nanostructured materials helps in reaching the most efficient electrode architectures, as discussed by Rolison [2]; however, achieving these results has proven difficult, and long-range ion diffusion in the electrolyte is still needed.

As an alternative to batteries, supercapacitors which are based on electrochemical reactions bridge the gap between batteries and standard capacitors [3]: they are capable of delivering much higher power rates, with lower specific energy than batteries but higher than capacitors. For certain applications, such as wireless sensor networks or RFID tags, having a small, possibly integrated, miniaturized energy reservoir capable of delivering high power densities represents a great benefit. Backup systems and dc-dc converters [4] are examples where high-density capacitors can leverage their advantages. Other applications for them include integrated circuit (IC) decoupling integrated on package. The trend to integrate passive components as near to the logic as possible provides an opportunity for high-capacitance devices.

Research in supercapacitors is a hot topic, with several research groups actively working in this area. Capacitors can be broadly classified in two groups: electrolytic capacitors (ELCs) and electrostatic capacitors (ESCs). Generally speaking, ELCs are based on the double-layer formation and offer moderate capacity densities at the expense of power density, parasitic equivalent series resistance (ESR) and operating frequency. On the other hand, ESCs have lower specific capacity but provide a much higher charge/discharge rate, lower ESR and higher operating frequencies. Electrochemical capacitors have some of the drawbacks inherent to electrolytic devices, mainly temperature and aging degradation and, to a lesser degree, a limited charge/discharge rate due to charge transport. To improve the diffusivity of electrons, 3-D structures have been proposed for high capacitance [1]. In this area, the development of nanotechnology has spurred research in supercapacitors. Many publications about nanomaterials applied to charge storage can be found going from exotic oxide electrolytes [5] to carbon nanotubes [6], nanospheres [7], graphene and others [8]. Although the use of new materials has mitigated the impact of the aforementioned problems, they are still present and impair the use of ELCs for certain applications.

In contrast, ESCs do not present the drawbacks of electrolytic capacitors, but in exchange, they have a much smaller specific capacitance (*C*_sp_). Two solutions are possible to regain some of the lost capacitance: dielectric choice and 3-D structuring to achieve a greater effective area. Regarding dielectric options, materials are classified as class 1 or direct/paraelectric and class 2 or ferroelectric [9]. Paraelectric materials have low to moderate dielectric constants (less than 100) while ferroelectric dielectrics may present giant dielectric constants (greater than 1,000). Examples of type 1 materials are silicon dioxide (SiO_2_) and alumina (Al_2_O_3_); type 2 material examples are barium titanate (BaTiO_3_) superlattices or lead titanate (PbTiO_3_). The right dielectric is dependent on the application. For type 2 materials, though they may seem the obvious choice, there are weak points to take into account: the most important is the dielectric constant behaviour, as it is a function of the applied electric field, frequency and temperature [9]. These materials are usually very sensitive to temperature, and humidity may also affect them. Aging may also be a concern. Obtaining the large promised dielectric constant for these materials can involve a complex fabrication process [10], as specific crystallinity may be needed and temperature can degrade the crystal structure and process variability can be high. On the other hand, class 1 dielectrics offer much more stable electrical parameters, although the aforementioned reduced dielectric constant. Therefore, to achieve competitive specific capacitance figures, a capacitor using class 1 dielectric must resort to effective area enhancement by taking advantage of the third dimension. This can be achieved by using stacked layer structures of alternating electrode-dielectric-electrode (multilayer ceramic capacitors (MLCC)) or by creating full 3-D geometries like macroporous silicon.

In recent years, nanoporous materials based on silicon and alumina [11-13] have been reported to have very large surface to volume ratios and proposed for high-capacitance devices. Reported devices show structures of pores with diameters typically below 200 nm with electrodes formed by conducting oxides or semiconductors such as titanium nitride (TiN) or aluminium zinc oxide (AZO) either single wall or multilayered, grown by deposition methods such as metal-organic chemical vapour deposition (MO-CVD) or atomic layer deposition (ALD). For these devices, series resistance can be elevated and may become an issue for certain applications. Furthermore, they require costly fabrication techniques and equipment. Attempts to reduce ESR and increase capacitance have led to the proposal of nanotubes, nanowires and nanospheres. Onion-like carbon nanospheres have been reported to achieve up to 2 and 17 nF/mm^2^[7]. These devices necessarily employ very thin oxides, generally ALD-deposited alumina, resulting in high leakage and reduced breakdown voltage. Metal-in-metal (MIM) devices have been reported to achieve up to 35 nF/mm^2^[14] and more recently near 1,000 nF/mm^2^[12], but with poor operating frequency. A remarkable MIM-like structure was presented in [15], where a rolled structure was obtained by self-rolling into a tight cylinder using stress forces of the different layers. This device showed a capacity density of 2,000 nF/mm^2^ and extended operating frequency. Its main disadvantage is the high surface required for the initial layout of the self-rolling layers, which may impair yields or feasibility.

Porous silicon (PS) was suggested for capacitive applications in [16] due to the potential increased surface, while the first macroporous silicon (MPS) capacitor was reported in [17] by Lehmann et al. several years later. MPS is a novel material mature enough as for several key industry players like Siemens [17] or NXP [18,19] are pioneering the use and adoption of PS and MPS for capacitors. For instance, the devices described in [17] use electrochemically etched (EE) silicon to obtain the MPS structures. This same technique is used by Roozeboom et al. in [20]. Reactive ion etching (RIE) is also presented as an alternative technique for fabrication of macroporous silicon. This technique was also employed in [20] to obtain 100-nF/mm^2^ devices. More recently, an improved device using MIM stacks and MPS was presented in [18] reaching up to 400 nF/mm^2^, though it used very thin alumina as insulator which negatively impacted leakage and breakdown. Using macroporous silicon opens up the possibility to use either ordered or random pore distribution. Random MPS has fewer fabrication steps, simplifying the design and reducing cost. Moderate specific capacitances about 25 nF/mm^2^ have been reported in [21], though the described capacitors have larger than expected leakage and high ESR. The main shortcoming of random macroporous silicon is the clustering effect of pore growth, creating areas with varying porosity which creates high-resistance current paths in dense areas and low-capacitance regions in sparse areas. Furthermore, sharp bends and spikes are common for random MPS, which can create electric field ‘hot spots’ and thinner oxide, which may result in insulator failure. Those problems can be avoided by using ordered MPS. Fabrication of ordered porous silicon is more complex but allows to obtain consistent and highly repeatable 3-D structures. Usually, standard ultraviolet (UV) lithography is applied to define pore location; an alternative way to obtain local-range ordered porous silicon is to use diblock copolymer self-assembly as presented in [22], in which fabricated devices with 31.3 nF/mm^2^ specific capacitance are reported.

In this paper, high-capacitance devices based on EE silicon have been fabricated and characterized. The presented devices are fabricated using 3-D MPS structures of ordered pores arranged in a square lattice of 4-μm pitch and diameter of 3 μm. The electrical parameters of the devices are enhanced by using highly doped substrates and metal electrodes. Fabrication advantages are leveraged to outline a simple and ready-for-mass-production method. A simple model is proposed for the device which yields accurate predictions and allows easy device design to meet application needs. The fabricated devices are comparable or better than equivalent EE devices found in literature, having high specific capacitance and better ESR.

## Methods

In this paper, the capacitance of a passive device based on a MPS trench structure is studied and modelled. Silicon is used to form a 3-D high surface-to-volume medium and to work as one of the capacitor electrodes. The insulator used for the devices was thermally grown silicon dioxide (SiO_2_). Several thicknesses were tested to assess electrical parameters and reachable capacitance. Specifically, the goal was to maximize capacity while keeping low the leakage current. Alumina was also tested as an insulator. Comparisons of ALD-deposited alumina to SiO_2_ were initially performed by measuring leakage. To obtain good frequency response and low ESR, the second electrode was formed by electrodeposited nickel. Electrical characterization was performed by impedance spectrometry and quasi-static *I*-*V* measurements. Structural analysis of the devices is performed by scanning electron microscopy (SEM) of selected samples.

### Macroporous silicon

**Figure 1:**
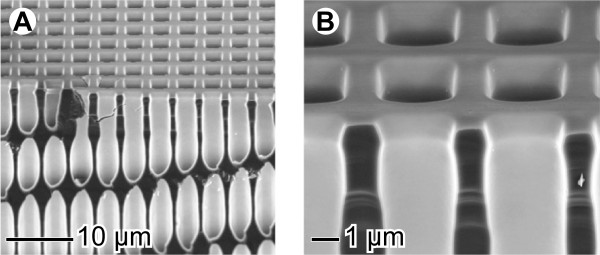
**Capacitors’ macroporous silicon microstructure.** Detailed SEM view of the macroporous silicon structure fabricated using electrochemical etching. **(A)** A general view of the etched pores: 3-μm-diameter cylinders and 240-μm depth. **(B)** A close-up view of the pores' head and the insulating SiO_2_ layer thermally grown.

The capacitor devices herein presented are designed to take advantage of the increased surface that is achievable using macroporous silicon fabricated by standard and micromachining methods. For the fabrication of the macroporous structures, the electrochemical etching method proposed and described by Lehman is applied. This method is extensively described elsewhere [23,24], and it will briefly described here. Silicon n-type wafers with <100> crystallinity are used to etch pores growing perpendicular to the wafer surface. To obtain stable and well-formed pores, the doping level of the wafer must be taken into account. In our case, for a 4-μm lattice pitch, a wafer resistivity around 3 Ω⋅cm is adequate. Pores are subsequently formed by anodic etching of silicon in hydrofluoric acid (HF). As described in [23], for the dissolution of silicon with fluorine, holes are needed, as the wafers used are n-type, and minority carriers must be generated somehow. Specifically following Lehman's method, holes are photogenerated by backside illumination of the porosified sample with an infrared (IR) light source. Etching speed can be controlled by adjusting both illumination (hole current) and biasing voltage, which allows creating modulated pore profiles [25]. Temperature and acid concentration also play a determinant role in etching speed; for the devices here presented, a 5% wt. HF at 15°C solution is used. To fabricate ordered MPS, a standard photolithography is used previous to EE to transfer the pore pattern. A square array with a 4-μm spacing is used for all capacitors. Etched pores have approximately 3 μm in diameter and are 240 μm deep. An example of the obtained MPS structure is shown in Figure 1. The ratio of the final structure surface to the original surface area can be defined as the area enhancement ratio (AER), which is defined as *AER* = *A*_tot_/*A*_cell_, where *A*_tot_ is the total surface of the unitary cell after etching and *A*_cell_ is the area of the unitary cell before etching. The etched area is a circular patch with a diameter about 18 mm.

### Membrane preparation

A porous membrane must be prepared from the just-etched MPS in order to be able to introduce the Ni inside the pores. As the etched pores do not reach the back face of the silicon wafer, a standard micromachining technique is employed to etch the excess silicon from the back face and reach the pore tips. This process is an alkaline etching of silicon in hot potassium hydroxide (KOH) with a 25% wt. concentration at 85°C. Prior to the KOH etch, a protective layer of SiO_2_ is grown to avoid rapid material dissolution once the alkaline bath reaches the pores. The etched area is limited to the porosified circle. After reaching the pore tips, the membrane is opened by stripping the protective oxide. To achieve good electrical conductivity in the silicon electrode, a phosphorous diffusion step is performed with POCL_3_. After drive-in, the doping level inside the pore walls is greater than 10^19^ cm^−3^ along the full interpore silicon.

### Insulator deposition

The insulating layer used for the devices was thermal silicon dioxide. Oxide was grown in a dry oxygen atmosphere in a high-temperature furnace at 1,100°C. Oxides ranging from 15 to 90 nm were grown and checked by ellipsometry. To obtain high-quality oxides, sample preparation is of utmost importance as defects increase with the enlarged surface; therefore, aggressive RCA cleaning was necessary for the samples. To improve capacity density, 30-nm ALD-deposited alumina was tried both on silicon and a 5-nm-thin supporting SiO_2_ layer. Nevertheless, leakage was in all cases too high and therefore alumina was discarded for the insulating layer.

### Nickel electrodeposition

**Figure 2:**
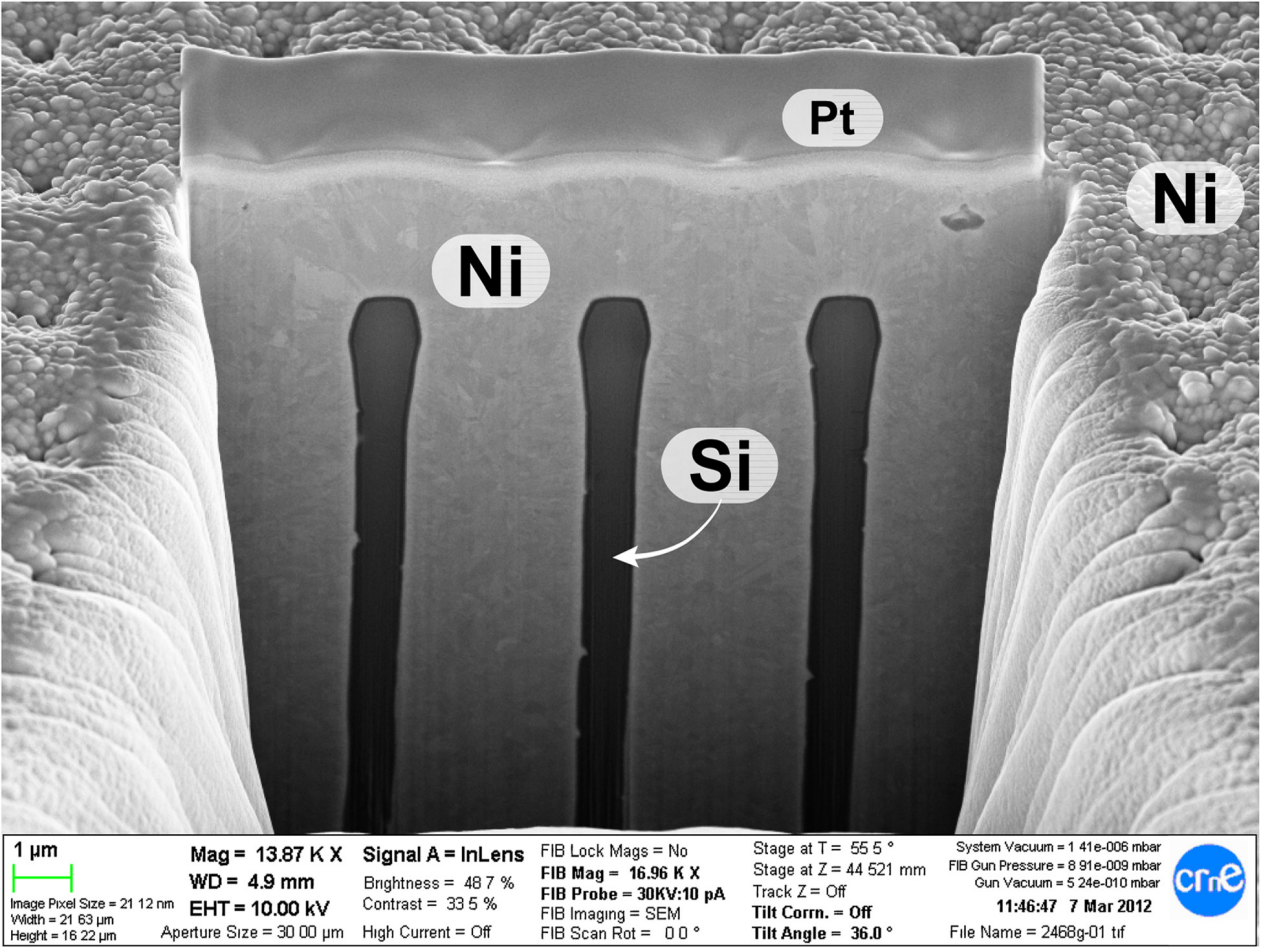
**Nickel electrode conformation.** SEM view of a FIB cut revealing the pore filling. After the membrane is created, the pores are completely filled with electrodeposited Ni. Good surface conformation is verified, leaving no voids between the insulating layer and the nickel electrode.

**Figure 3:**
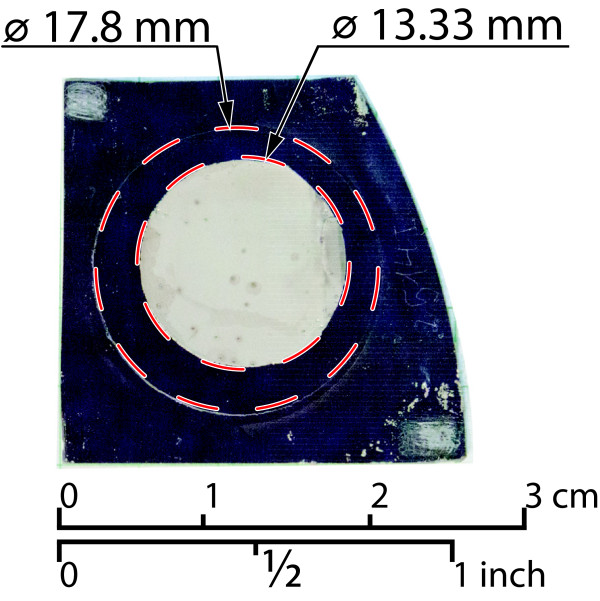
**Fabricated device.** Photograph of a finalized device. The porous area is a circular region of about 17 mm diameter, though the filled area is smaller. This is to avoid the borders where defects concentrate.

Once the membrane with the insulator is prepared, pores are filled with nickel using an industry standard electroplating technique. Firstly, an electrical contact is deposited in one of the faces by evaporation. The sample is afterwards placed with the metallized face covered in a nickel sulfamate bath at 50°C. The exposed face soaks the sample through the pores. Using a Ni counter electrode, a current is circulated through the metallized face promoting the precipitation of Ni and growth of a solid conformal nickel wire inside the pores. As can be seen in Figure 2, the resulting nickel wires completely fill the pores leaving no voids. Deposition rate is proportional to current and empirically measured. The total pore fill is therefore controlled by deposition time and current.The device is finalized by adding the contacts to each electrode, and the resultant device can be seen in Figure 3.

### Model and characterization

**Figure 4:**
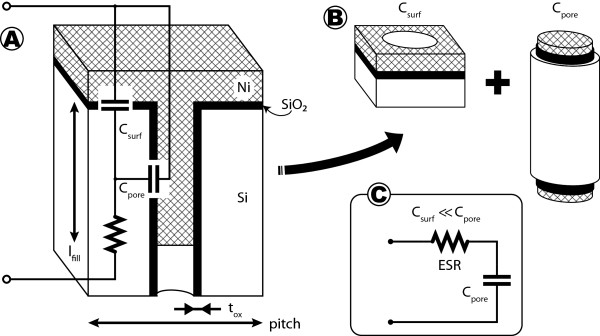
**Device unitary cell diagram.** Schematic representation of a single cell detailing the elements and the model extracted. **(A)** A section view of a unit cell is depicted, showing the different elements composing the unit capacitor element. Furthermore, the dimension definitions are shown in this sketch, along with a first electrical model. **(B)** A concept of the capacitances defined by the structure. Since *C*_surf_ is much smaller than *C*_pore_, the model reduces to a simple RC network, which is shown in frame **(C)**.

Inspecting the geometry of a unitary cell (see Figure 4), an initial model for the capacitance of the device can be derived. The capacitance for a single unitary cell is approximated as the capacitance due to the pore in parallel with the capacitance with the remaining top surface. As pores are nearly cylindrical, the pore capacitance can be equated to a cylindrical capacitance of length that of the nickel wire. It is possible to demonstrate that the pore capacitance is much larger than the surface; therefore, to simplify calculations, one can choose to ignore the small surface capacitance. Given that the nickel fills a given depth not necessarily the whole pore length and may not cover the full porous surface, we can define an *effective* AER (AER′) which takes into account the area of the nickel deposit: AER^'^ = *A*_Ni_/*A*_cell_. The fabricated devices have an AER′ around 83.

Electrical characterization is performed by impedance spectroscopy (IS) scanning a frequency range from 40 Hz to 1 MHz using a 50-mV signal using an Agilent 4294A impedance meter (Agilent Technologies, Inc., Santa Clara, CA, USA). Voltage biasing at 1 kHz is also measured with the 4294A from −10 to 10 V. Leakage is measured with an Agilent 4156C device analyser with a 1-V test voltage.

## Results and discussion

Several macroporous silicon membranes were prepared using the EE technique for the pore fabrication and local KOH etching for the membrane opening. By using the etching conditions mentioned above, square lattices of 4-μm pitch and 3-μm pores were obtained. The pore lengths were designed to be of 240 μm. As mentioned, macroporous silicon fabricated using the EE technique has good geometry uniformity and is very repeatable. This has been confirmed with SEM analysis of some samples, resulting in pore diameters within a few nanometres and lengths about a few microns off of the desired values (see for instance Figure 1). Pore wall smoothness and profile are also good. Pore defects, such as branching or pore death, are present near the border of the etching zone, extending less than 1 mm inside the porous area. For this reason, we may want to avoid using the full extent of the porosified material to perform the nickel electrodeposition. Actual structure dimensions change slightly after the membrane is opened due to the oxidation and oxide stripping steps performed, as well as the alkaline etching. In particular, pores widen about 80 nm, and length can shorten up to 10 μm. After the nickel electrodeposition, SEM analysis confirms that the tubes are filled without voids, as shown in Figure 2.

Several capacitors based on MPS with different oxide thicknesses and materials were measured. It was found that for 15 nm SiO_2_ and for ALD alumina, leakage current was too high, resulting in a few microamperes around 2 μA at 1 V test voltage, and thus a parallel resistance of 500 kΩ or less. These figures were found to be too high for what was expected. SEM studies of the failed capacitors did not reveal a consistent failure cause. In some instances, oxide pinholes were detected, although in other cases, no apparent damage was seen. A possible explanation is that defects in oxide were caused by either contamination during fabrication or the presence of microporous silicon which after oxidation gave rise to low-quality oxide. Nevertheless, no satisfactory explanation was found for the failure of the ALD-alumina-coated samples.

**Figure 5:**
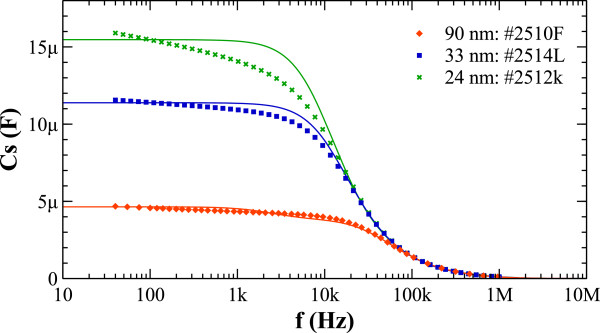
**Frequency response of the capacitors.** Frequency response for capacitors with different insulator thicknesses. Measurements were taken with a 50-mV excitation and a logarithmic frequency sweep from 40 Hz to 1 MHz.

However, for the thicker samples, starting from 24-nm-thickness silicon dioxide, leakage was reduced to acceptable levels below 1 nA, yielding a best case about 0.2 nA/μF·V. Given the high leakage resistance, a simple model for a capacitor using only an R-C element is enough to accurately describe the electrical behaviour of the MPS capacitors. We report the extracted parameters for samples with 24-, 33-, and 90-nm SiO_2_ insulating layers. After frequency-resolved IS was performed on these samples, data was fitted by a non-linear least squares method to the model, as can be seen in Figure 5. The results are summarized in Table 1. The good agreement of measured parameters with the theoretically derived values for capacitance can be appreciated. As can be readily observed, the highest specific capacitance is obtained for the 24-nm-thickness insulator. A notable aspect of the fabricated devices is the low ESR near 1 Ω for all devices. This almost constant residual ESR is attributed mainly to the silicon electrical path: the nickel electrode has a much lower series resistance which accounts to less than 1 mΩ, while the silicon current path is narrower, as is mainly located near the surface of the silicon walls, and has higher resistivity (about 15 mΩ·m.) This imposes a trade-off when designing the capacitors. On one hand, to increase capacitance, a possible way is to use a closed packed lattice and a smaller lattice pitch, but this will result in narrower silicon walls and therefore larger ESR. On the other hand, a different interconnect for the silicon electrode may slightly improve this figure. Furthermore, an alternative isolation material may be considered depending on the application, although leakage could be a concern. Thanks to the low ESR, the fabricated MPS capacitors are able to operate up to 10 kHz as seen in Figure 5. Finally, there is the option of completely removing the silicon electrode and replacing it with another metal. This is a technologically complex operation which may not be economically justified.

**Table 1:** Device characteristics and electrical parameters

**Ox. thick**	**Fill**	**Theory**	**Model**		
*t*_*ox*_ (nm)	*L*_*fill*_ (nm)	*C* (μF)	*C* (μF)	ESR (Ω)	*C*_*sp*_ (nF/mm^2^)
90	150	5.09	4.64	1.08	36.4
33	130	11.67	11.39	1.12	83.4
24	130	15.97	15.48	1.09	109.7

The obtained results can be compared with those from Roozeboom et al. [20,26], who used the EE technical as well as DRIE to obtain similar-sized ordered macroporous silicon structures. They report specific capacitances up to 100 nF/mm^2^, but total device capacitance only up to 1 μF. In their work, the dielectric is a 30-nm-thick oxide-nitride-oxide (ONO) stack; therefore, as expected, the *C*_sp_ is similar to the values here reported. Instead of a metal electrode, they use the more common n-type doped poly-Si-deposited layer. The lower total capacitance is explained because of their choice of geometric layout. A relevant figure is the low 0.1-Ω ESR, but these figures are reported for 10-nF devices. In particular, the devices presented in Roozeboom et al.'s work are intended for RF operation and have very low values, allowing to operate up to high frequencies. For the devices presented in this paper, emphasis is made on energy storage applications.

A more recent work using macroporous silicon can be found in Sancho et al. [27]. In this work, disordered macroporous silicon is used, with pore sizes similar to the ones here presented, but shorter pore length. In Sancho et al.'s work, a thick silicon dioxide layer and the poly-Si is used as the counter electrode. The planar surface is normalized to 1 cm^2^. These values explain the low capacitance figures about 4 nF/mm^2^. Furthermore, series resistance is very high resulting in a poor frequency response of less than 100 Hz. Nevertheless, the use of a thick oxide allows the use of the devices for high-voltage applications, although this is not mentioned in Sancho et al.'s work.

Higher specific capacitances are reported in Klootwijk *et al.*[18], where values up to 440 nF/mm^2^ are demonstrated. The devices shown in this paper used a multilayer MIM stack using a slightly denser macroporous silicon substrate as a frame. To obtain such devices, a complex fabrication set-up is needed, using high-temperature processes. They report leakage figures similar to those of this work, but breakdown voltages up to 7 V. The reported ESR is around 10 Ω.

One aspect to draw attention into is the smaller pore fill in respect to pore length. As it can be seen in Table 1, the nickel electrode does not fully penetrate the MPS structure. This is due to the fact that oxide quality is worse near the pore ends and therefore should be avoided. Ideally, the pores present very good surface conditions and oxide should be uniform, but there exists the suspicion that post-etching processes like KOH etching and diffusion may introduce defects to the surface of the pores which affect most the tip of the pores. With a good controlled insulator, it should be possible to take advantage of the total pore length.

**Figure 6:**
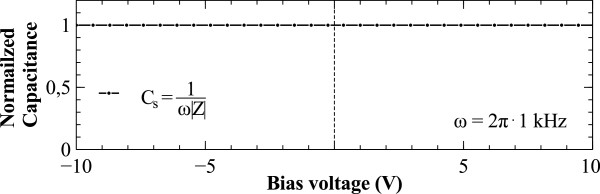
**Bias sweep of the capacitors.** The normalized capacitance of a test capacitor is checked at 1 kHz constant frequency and voltage bias ranging from −10 to 10 V. Bipolar behaviour can be observed along with no MOS inversion layer effect.

Bias voltage sweeps have been performed for some of the devices, which were capable of withstand up to 10-V voltages across plates as can be seen in Figure 6 and short-term burst up to 20 V. Because the devices are electrostatic capacitors and class 1 dielectrics are used, the devices show symmetric bipolar operation. Even more, as the silicon electrode is very highly doped, no inversion layer effect is noticeable as is typical for CMOS capacitors. For the 24-nm devices, this allows to a breakdown voltage close to 8.3 MV/cm, which is near the theoretical value for silicon dioxide. The values obtained compare favourably to the work by Roozeboom et al. [20,26], Sancho et al. [27] and Klootwijk et al. [18].

## Conclusions

In this paper, high-capacity MPS capacitors are presented using macroporous silicon as one of the electrodes and an electrodeposited nickel counter electrode. A high specific capacitance up to 110 nF/mm^2^ has been obtained for a 24-nm insulator layer of thermal silicon dioxide. Low leakage is achieved and high-voltage bipolar operation is possible. A notable low ESR is obtained around 1 Ω, limited by the silicon electrode, and some improvement paths are explored. Moreover, the absence of MOS capacitor behaviour makes these devices suitable for large signal operation. The fabrication techniques are simple and require few or no specialized equipment and few high-temperature steps. Furthermore, a simple model for the devices has been obtained which allows designing the capacitors according to specifications with good accuracy. The small footprint of these devices may allow them to be integrated on package and eventually on die. Finally, thanks to the high-frequency operation, the devices are currently aimed for IC decoupling applications and dc-dc converters. By careful design, they may be suitable for energy storage applications.

## Abbreviations

AER: area enhancement ratio; AER′: effective AER; ALD: atomic layer deposition; AZO: aluminium zinc oxide; EE: electrochemical etching; ELC: electrolytic capacitor; ESC: electrostatic capacitor; ESR: equivalent series resistance; IC: integrated circuit; IR: infrared; IS: impedance spectroscopy; MIM: metal-in-metal; MLCC: multilayer ceramic capacitor; MO-CVD: metal-organic chemical vapour deposition; MPS: macroporous silicon; ONO: oxide-nitride-oxide; PCB: printed circuit board; PS: porous silicon; RFID: radio frequency identification; RIE: reactive ion etching; SEM: scanning electron microscopy; UV: ultraviolet.

## Competing interests

The authors declare that there are no competing interests.

## Authors’ contributions

DV participated in the concept and design of the devices and studies, provided support along the fabrication process and carried out measurements and data analysis and fitting. JR worked in the fabrication process, especially in correction of leakage behaviour; developed the final fabrication steps; and participated in the devices' characterization. FM helped in measurements. RP worked in the fabrication process tuning and first prototype fabrication and measurements. AR conceived the study and helped in data analysis, and contributed to coordinate and draft the manuscript. All authors read and approved the final manuscript.

## Authors’ information

DV graduated in telecommunications engineering from the Universidad Politécnica de Cataluña (Spain). He is currently pursuing his doctoral research in macroporous silicon and its applications. He is also working in photonic crystals and sensors. AR graduated in telecommunications engineering from the Universidad Politécnica de Cataluña (Spain). From 1987 to 1992, he worked at IMEC (Belgium) towards his doctoral work in the field of Polysilicon Thin Film Transistors. In 1993, he became an associate professor in the Escuela Técnica Superior de Ingenieros de Telecomunicación de Barcelona. Dr. Rodríguez has worked in solar cells, bipolar transistors, polysilicon thin film transistors and MEMS. Concerning MEMS, he has worked in flow sensors, accelerometers, different kinds of artificial noses based in metallic oxides or polymer resonating structures, RF MEMS and MEMS actuators. At present, Dr. Rodríguez is active in the fields of porous-silicon-based MEMS/MOEMS, photonic crystals and Bio-MEMS.
